# Perspective: Teaching Kitchens: Conceptual Origins, Applications and Potential for Impact within Food Is Medicine Research

**DOI:** 10.3390/nu15132859

**Published:** 2023-06-24

**Authors:** David M. Eisenberg, Lorena S. Pacheco, Auden C. McClure, John W. McWhorter, Kate Janisch, Jennifer Massa

**Affiliations:** 1 Department of Nutrition, Harvard T.H. Chan School of Public Health, Boston, MA 02115, USA; lpacheco@hsph.harvard.edu (L.S.P.); kjanisch@hsph.harvard.edu (K.J.); jmassa@hsph.harvard.edu (J.M.); 2 Dartmouth Geisel School of Medicine, Hanover, NH 03755, USA; auden.c.mcclure@hitchcock.org; 3 Suvida Healthcare, Houston, TX 77039, USA; j.wesley.mcwhorter@gmail.com

**Keywords:** culinary medicine, culinary nutrition, cooking, integrative nutrition

## Abstract

There is a need to identify innovative strategies whereby individuals, families, and communities can learn to access and prepare affordable and nutritious foods, in combination with evidence-based guidance about diet and lifestyle. These approaches also need to address issues of equity and sustainability. Teaching Kitchens (TKs) are being created as educational classrooms and translational research laboratories to advance such strategies. Moreover, TKs can be used as revenue-generating research sites in universities and hospitals performing sponsored research, and, potentially, as instruments of cost containment when placed in accountable care settings and self-insured companies. Thus, TKs can be considered for inclusion in future health professional training programs, and the recently published Biden–Harris Administration Strategy on Hunger, Nutrition and Health echoes this directive. Recent innovations in the ability to provide TK classes virtually suggest that their impact may be greater than originally envisioned. Although the impact of TK curricula on behaviors, outcomes and costs of health care is preliminary, it warrants the continued attention of medical and public health thought leaders involved with *Food Is Medicine* initiatives.

## 1. Introduction

In 2020, the United States (US) spent USD 4 trillion on health care [1] and USD 1.7 trillion on food [2]. As suboptimal dietary patterns are associated with the majority of the leading causes of death in the US [3] and globally [4], there is now a heightened urgency to identify novel strategies whereby individuals, families and communities can learn how to access and prepare affordable and nutritious foods in combination with evidence-based guidance about diet and lifestyle to help improve health status while addressing rising health care costs and issues of nutrition insecurity. Importantly, such strategies need to consider both personal habits and societal pressures using a combination of reproducible, experiential learning programs in association with a robust research network. Teaching Kitchens (TKs) routinely include nutrition education, hands-on cooking instruction, mindfulness training, exercise prescription and motivational interviewing [5]. TKs can be considered multidisciplinary strategies with the potential to improve health outcomes.

Further, TKs have the potential to serve as a conduit in addressing diet-related social determinants of health through a variety of food and nutrition initiatives that often include culinary medicine education paired with food prescriptions. These TK-related programs can provide a pathway for clinic-to-community partnerships that amplify the diversity of nutritious foodways and flavors while creating a safe atmosphere for peer-to-peer interactions and practical food-based education to overcome common barriers to healthy eating.

In this perspective paper, we detail the basic principles of TKs, their conceptual origins, preliminary evidence of effectiveness, recent growth, and potential for impact as educational classrooms and translational research laboratories.

## 2. Teaching Kitchens

In recent decades, nutrition scientists have determined which foods (and nutrients) are associated with heightened or diminished risks of chronic disease [6,7]. Additionally, clinical research has established that genes are “not destiny”, and that epigenetic factors, including how individuals choose to eat and exercise, impact their disease risk apart from their genetic predisposition [8,9]. However, we now recognize that social determinants of health, along with an emergence of an “obesogenic culture”, have contributed to increasing rates of obesity, pre-diabetes, and diabetes worldwide [10,11]. Additionally, the percentage of meals consumed outside one’s home has increased significantly over the past 40 years [12] and eating at fast food restaurants has been shown to add substantially to chronic disease risk and increased mortality [13,14]. Additionally important, and less well-known, is the fact that most medical schools do not require education in or competencies relating to advice to patients about nutrition and lifestyle as prerequisites for licensure. This limits direct patient recommendations from practicing physicians, as well as referrals to specialists such as Registered Dietitians (RDNs) and Certified Nutritional Specialists [15,16]. This lack of required nutrition education among non-RDN health professionals has recently gained the attention of Congress. In May 2022, the US House of Representatives passed the bipartisan McGovern Resolution (Resolution 1118 [17]) calling for medical schools, graduate medical education programs and other professional training programs to provide meaningful physician and health professional education on nutrition and diet or risk the discontinuation of USD 10.3 billion in federal funding for the training of future health professionals. The recent White House National Strategy on Hunger, Nutrition and Health also includes similar language in its “Call to Action” [18].

### 2.1. Defining Teaching Kitchens: Conceptual Origins and Core Components

The conceptual origins of modern-day TKs relate to tenets of Traditional Chinese Medicine (TCM). The two main principles, attributed to China’s first emperor, Huang Di, are: (1) prevention is always superior to intervention; and (2) the ways we eat, move and think (control our thoughts and emotions) predictably influence our health and determine our recuperative capacity [19].

These insights, codified by Chinese authors 24 centuries ago [19], promote the idea that optimal health and wellbeing are more likely to occur if one learns how to eat, move and think more healthfully. A TK can be viewed as a contemporary representation of this East Asian strategy to maintain and optimize health and wellness. TKs are physical or virtual venues which include more than kitchens; where individuals come together to learn life-enhancing and health-promoting skills, knowledge and strategies through experiential learning involving food.

The comprehensive and essential evidence-based educational components of TKs include: (1) nutrition facts based on the latest science, i.e., what to eat more of or less of and why; (2) hands-on culinary instruction to teach health care professionals how they, and their patients, can prepare healthy, delicious, easy-to-make, affordable and sustainable recipes and meals; (3) the importance of movement and exercise apart from the importance of a healthy diet; (4) mindfulness techniques to apply when selecting foods, cooking, eating, appreciating levels of satiety and learning to “be present” as part of daily life and work; and (5) sustained behavior change informed by motivational interviewing and health coaching principles.

As such, TKs go beyond instruction in culinary skill acquisition. They represent “learning laboratories” where individuals and families learn information and practical skills to promote well-being and prevent or manage chronic disease.

### 2.2. The Impact of the Annual “Healthy Kitchens Healthy Lives” [20] Conference

In 2006, Harvard University and The Culinary Institute of America launched the annual continuing medical education conference entitled Healthy Kitchens Healthy Lives (HKHL; “www.healthykitchens.org”, accessed on 10 May 2023) [20,21]. This conference has been offered annually and has provided nutrition and lifestyle educational updates to more than 7000 health professionals [21]. At the 2015 HKHL conference, more than 100 (out of 400) registrants reported that they had built or would soon build a TK for the purpose of educating patients, health professionals and/or employees at their respective home institutions. The unanticipated response by attendees led to the establishment of the Teaching Kitchen Collaborative (TKC) [22], an invitational collaborative of 49 organizations with TKs in the US, Canada, Italy, Germany and Japan, which includes hospitals, medical schools, health care delivery systems, K-12 schools, colleges, universities, corporations, YMCAs, Veteran’s Administrative settings, public libraries and botanical gardens as members. All members are committed to developing best practices and a research network whereby the impact of TK-related programs can be rigorously evaluated and refined across settings and populations [5].

### 2.3. Preliminary Evidence of the Effectiveness of Teaching Kitchens

Although there have been a number of studies on culinary medicine interventions [23], there have been a smaller number [24,25,26] evaluating the impact of TK curricula. There is a growing body of evidence pointing towards the positive associations between health and wellness outcomes and Food Is Medicine [27]. However, the specific intervention of TKs has been smaller and highly heterogeneous regarding the intensity, duration and depth of component information and teaching in these interventions [28], making it difficult to properly systematically review and meta-analyze the diverse existing published literature. Nonetheless, we found three studies suggesting that participation in a TK intervention program improved clinical outcomes. One study [24] involved employees (*n* = 39) of a self-insured educational institution (Hyde Park, NY, USA), and addressed the five TK components over 16 weeks. Investigators reported significant (*p* < 0.05) mean [SD] changes from baseline to 6 months for weight (−4.2 kg [6.5]), systolic blood pressure (−10.08 mm Hg [119.07]), diastolic blood pressure (−8.24 mm Hg [11.72]) and waist circumference (−3.24 in [3.09]). Another study [25] included patients (*n* = 72) with type 2 diabetes (Montreal, QC, Canada) and an elevated cardiovascular risk who were provided with culinary skills, eating behavior and nutrition and promotion of physical activity for 24 weeks, and findings included improvements in weight (mean change −2.2%; 95% CI −3.6 to −0.8) and HbA1c (mean change −0.3% HbA1c, 95% CI −0.6 to −0.1), as well as suggestion of systolic blood pressure reduction (mean change −3.5 mm Hg, 95% CI −7.8 to 0.9). Researchers also noted beneficial changes in eating control (11.2-point Weight Efficacy Lifestyle score change, 95% CI 4.7 to 17.8) and step counts (mean change 869 steps/day, 95% CI 198 to 1540). The third study [26] involved patients with an elevated cardiovascular risk (*n* = 429) (Lyndhurst, OH, US) in an intensive 6-week (8 h per week) course which integrated nutrition, physical activity and stress management educational elements, followed by three 4 h sessions over 6 months. At week 30, there were significant (*p* < 0.05) mean [SD] changes in weight (−6.8 kg [SD 6.9]), waist circumference (−6.1 cm [7.3]), glucose (−4.5 mg/dL [29.6]), triglycerides (−26.4 mg/dL [58.5]), low-density lipoprotein cholesterol (−7.9 mg/dL [25.1]), HbA1c (−0.2% [0.64]), insulin (−3.8 microU/mL [11]), ultra-sensitive C-reactive protein (−0.9 mg/dL [4.8]) and high-density lipoprotein cholesterol (+3.7 mg/dL [8.4]). In addition to biometric and laboratory measurements, all three studies documented that TK curricula can be feasibly applied to a range of settings and these have the potential to favorably alter behaviors, biomarkers and clinical outcomes [24,25,26].

Additionally, there have been studies examining the impact of TK educational programs on medical students [29,30,31]. These have suggested that hands-on culinary instruction in combination with nutrition education improves medical students’ knowledge and counseling of dietary patterns. Two other studies reported benefits of a medical school-based TK on diet quality in a sample of adults with low literacy [32], and cardiometabolic biomarkers in patients with T2D [33]. Additionally, a recent trial assessed the effect of a culinary education curriculum on Mediterranean diet adherence and food cost savings in families in New Orleans. The authors estimated that this kitchen-based education saved families USD 21.70 per week [34].

### 2.4. Clinical Case Study

The following is a representative case study involving an adult with enhanced cardiovascular risk, who benefited from a 6-week TK educational intervention:

PS is a 57-year-old environmental justice organizer with a past history of sleep apnea, hypertension, hypercholesterolemia, type 2 diabetes, cardiovascular disease and cardiomyopathy, referred to the Fresh & Savory TK Shared Medical Appt Culinary Medicine Program (based at MedStar Health in Washington, DC, USA) by his cardiologist.

He was a former long-distance runner and high school track athlete, who continued an active lifestyle and somewhat healthy diet into adulthood. Due to the tragic fatal accident of his 19-year-old talented son (premier ballet dancer), PS slipped into a clinical depression, gaining almost 140 pounds (165 to 304 pounds) in 3 years. He stopped jogging, stopped taking long hikes with friends in Rock Creek Park and began following a diet rich in ultra-processed “comfort foods”. During this 3-year period, he developed type 2 diabetes with a HbA1c of 10%, poorly controlled hypertension, hypercholesterolemia, insomnia, severe obstructive sleep apnea, cardiomyopathy with EF-41%, loss of mobility, new onset peripheral edema and dyspnea on exertion. He became dyspneic after walking 10–15 min.

At the time of his follow-up visit with his PCP, his vitals were as follows: height 5′7″; weight 298 pounds; body mass index 46.84 kg/m^2^; and blood pressure 194/110 mmHg. His PCP provided the standard of care: atorvastatin 20 mg/aspirin 80 mg/valsartan 320 mg chlorthalidone, 25 mg/carvedilol 12.5 bid/hydralazine 100 mg bid/metformin. He was instructed to lose weight and advised to limit “junk food”. He was referred to an endocrinologist.

After receiving his diagnosis of type 2 diabetes, PS gradually increased exercise to 30 min 5 days/week and started reading diabetes management and healthy-diet-related literature provided by his health care team. He lost 20–30 pounds but continued to feel confused about appropriate nutrition and felt considerable shame regarding multiple chronic conditions that he felt that he caused.

PS was referred by his cardiologist to the Fresh & Savory Shared Medical Appointment TK Program at MedStar Health. This consisted of six weekly 2 h sessions of culinary lifestyle skill building taught by a multidisciplinary team which included: an MD, chef educator, RD and medical student volunteers. PS enjoyed creating whole-grain dishes (aromatic quinoa), using new spices and powering up his plate with vegetable dishes. He shared new dishes with his wife and neighbors in their co-op. Journaling successes with SMART goals motivated him to expand his cooking skills. He was also encouraged to increase his workout routine. He added weight training 2 days per week and increased walking to 60 min 3 days per week. He noted that feeling the support of the Fresh & Savory team/community and achieving the culinary- and lifestyle-related goals he was learning increased his positive “self-talk”. He continued to meal plan, prep and exercise after the conclusion of the 6-week Shared Medical Appointment TK Program.

Six months after he completed the TK educational intervention, his HbA1c decreased to 5.8%. Metformin was prescribed by his medical team. His ejection fraction increased from 41% to 55–60%.

He continues to cook meals rich in whole grains, fiber, vegetables and legumes. He performs regular exercise, including working with a trainer twice weekly, meditation and yoga. His blood pressure is consistently under 130/80 mmHg, and he now remains free of dyspnea on exertion and free of any chest pain.

Current vitals (two years after enrolling in the TK Program): blood pressure 103/66 mmHg; weight 214 pounds; body mass index 33.50 kg/m^2^. Laboratory values include: high-density lipoprotein cholesterol 44 mg/dL; triglycerides 82 mg/dL, low-density lipoprotein cholesterol 72 mg/dL; total cholesterol 132 mg/dL; and HbA1c 5.8%.

This provides an example of how a series of TK classes can have life-changing effects on individual patients and their families. A video interview of this patient is publicly available at: https://www.facebook.com/MedStarHealth/videos/183703819644756/ (accessed on 10 June 2023).

### 2.5. Evidence of Growth in Teaching Kitchens, Teaching Kitchen Research and the Food Is Medicine Map [35]

Harvard University, with grant support from the National Institutes of Health (NIH), has hosted three Teaching Kitchen Research Conferences (TKRC; www.tkresearchconference.org, accessed on 10 May 2023): 2018 [36], 2020 [5] and 2022 [37]. Original research presented at the 2020 TKRC included studies involving TKs as applied to a range of target populations in educational, research and clinical care settings. The 2020 TKRC, held virtually, attracted more than 2500 registrants from 75 countries, 900 of whom confirmed the existence of a TK within their home organizations. The 2022 TKRC [37], held in-person and virtually, attracted more than 500 attendees where more than 300 came from institutions with TKs.

The recently released Food Is Medicine Map [35] invites Food Is Medicine-based organizations, including: TK Programs, Medically Tailored Meal Programs, Produce Rx Programs, Nutrition Incentive Programs and Fresh Food Farmacy Programs [38] to share information about the location and programmatic content of their respective organizations. The co-creators of this Food Is Medicine Map share the goals of (a) informing the public about these programs in their respective geographical areas; and (b) using this map to identify areas where multiple food-related stakeholder groups are co-located, thereby pinpointing “hot spots” where the potential for future collaborative research can be advanced in an effort to understand whether access to healthy foods, when coupled with emerging educational programs, may lead to positive changes in behaviors, biomarkers, clinical outcomes and costs. As such, TK educational teams are essential protagonists within the larger Food Is Medicine community; a group focused on educating individuals on more than what to eat more of and less of and why; or, how to prepare more nutritious foods, but also on realistic processes to incorporate affordable and culturally relevant options into their day-to-day lives.

### 2.6. Variations in Teaching Kitchen Design and Teaching Kitchens of the Future

**Figure 1:**
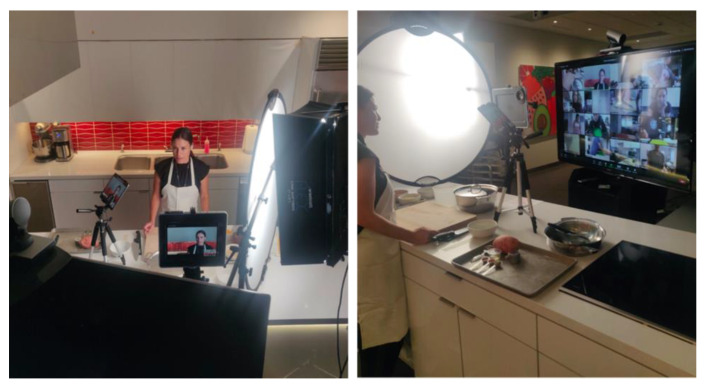
Variation in Teaching Kitchen design: virtual, interactive culinary training. ©David M. Eisenberg, MD.

A recent publication proposed the design of future TKs within health care and non-health care settings [39]. The authors provide an overview of TK designs, including those which only offer culinary demonstrations, offer both demonstrations and hands-on cooking training, involve built-in vs. portable (i.e., “pop-up”) facilities, offer in-person only education, provide virtual, interactive training only and those capable of either in-person and/or virtual sessions which use smartphones or tablet cameras aimed at the culinary instructors and trainees’ hands and faces [39] (Figure 1). In particular, “pop-up” TKs can be essential in community settings lacking a teaching space, as well as in clinics and hospitals that have established programs combining Produce and/or Food Rx Programs, food pantry access and culinary medicine curriculum [40].

**Figure 2:**
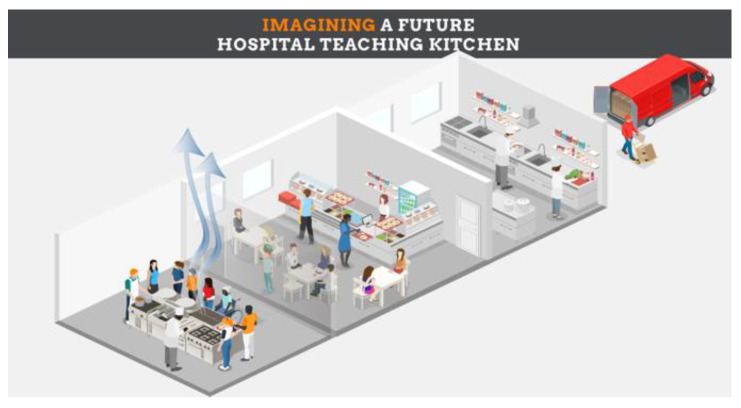
Future Teaching Kitchen using a ventilated ceiling in a hospital cafeteria setting. ©David M. Eisenberg, MD.

**Figure 3:**
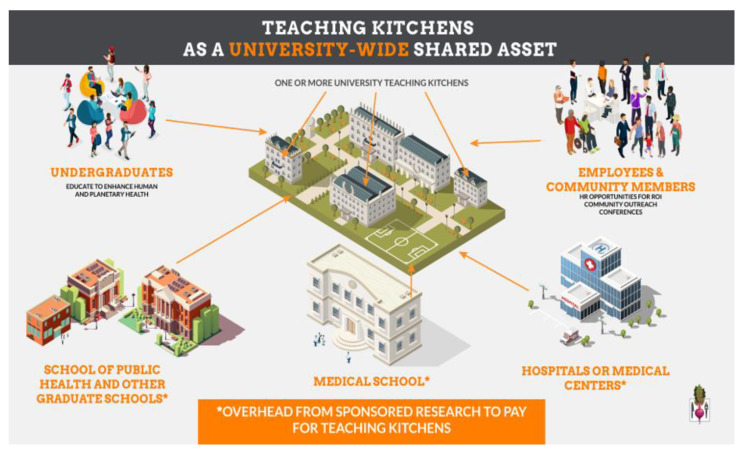
Beneficial impact of Teaching Kitchens as “shared assets” of a university with access to multiple stakeholder groups (e.g., students, patients, researchers, employees, etc.). ©David M. Eisenberg, MD.

Authors include a description of a futuristic TK design which can be incorporated into a new or existing cafeteria, using that building’s air handling units for silent ventilation (Figure 2) [39]. Additionally, such TKs can be used as clinical research sites for the conduct of sponsored research, thereby offering the opportunity to pay for the construction and maintenance of TK facilities within hospitals and universities (Figure 3). These can be accessed by students, researchers, health professionals and employees. This same approach may provide opportunities for private organizations to explore the impact of TK programs on the health, wellbeing, productivity and health-related costs of its employees. As such, TKs have the potential to serve as both educational classrooms and as research laboratories, and can generate research overhead and evidence of cost savings to offset costs associated with construction and maintenance.

### 2.7. Teaching Kitchens’ Relevance to Culinary, Lifestyle, Integrative and Conventional Medicine

**Figure 4:**
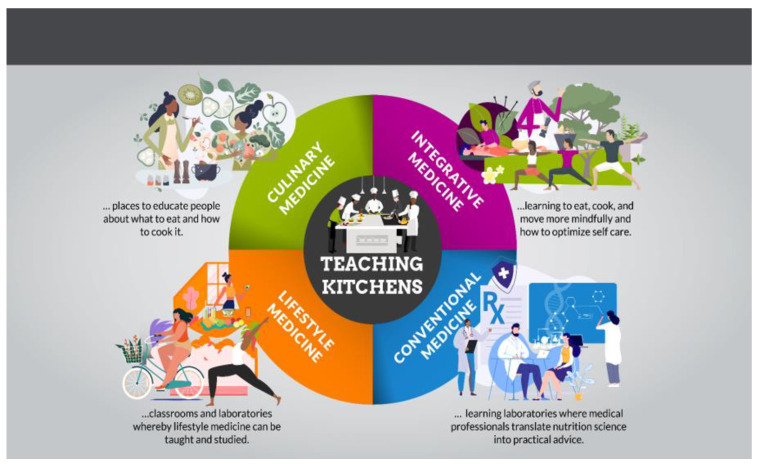
Teaching Kitchens in relationship to (1) culinary, (2) lifestyle, (3) integrative and (4) conventional medicine. ©David M. Eisenberg, MD.

TKs have been mentioned in the context of culinary, lifestyle, integrative and conventional Medicine, including use by RDNs (Figure 4). However, what has been lacking is a discussion about, and future consensus regarding, the optimal use of TKs as applied to each of these communities and settings.

TKs include ***Culinary Medicine*** information and skills as essential components, but also include additional educational goals which relate to optimal self-care above and beyond food, cooking and diet. TKs are intended to enable people to eat, cook, move and think more healthfully, as well as develop social connections and enhanced self-efficacy; whereas ***Culinary Medicine*** focuses primarily on nutrition facts and cooking skills, without additional instruction in the other areas mentioned above.

TKs can function as educational spaces whereby knowledge relating to ***Lifestyle Medicine***, including diet, cooking, exercise, mindfulness and behavior change can be shared; and trainees can have access to experiential learning in these domains. Several medical schools have robust initiatives in ***Lifestyle Medicine*** as interest in this discipline is rapidly expanding [41].

***Integrative Medicine*** includes several components of TKs such as nutrition, exercise, mindfulness and strategies to promote health-enhancing behavior change. Not surprisingly, ten current TKC members oversee ***Integrative Medicine*** Programs across the US. These include Northwestern Medical School, Cleveland Clinic, Medstar Health/Georgetown University School of Medicine, Harvard University; University of Minnesota; UCLA; University of Cincinnati College of Medicine, Hackensack Meridian Healthcare; Boston University Medical Center; University of Vermont, the University of Utah, Case Western Reserve and others. Additionally, the NIH’s National Center for Complementary and Integrative Health has been a co-sponsor of all three TKRCs. As such, TKs are of interest to the ***Integrative Medicine*** community and can be utilized for both educational and research purposes.

Since TK research often involves the evaluation of novel educational interventions for their impact on behaviors, biomarkers, anthropomorphic assessments, metabolites and microbiota patterns, genetic regulation, clinical outcomes and costs of care, they can be viewed as “clinical research sites” for ***Precision Nutrition*** research teams [5]. Novel methods of testing an individual’s response to dietary patterns and specific foods as they affect the gut biome and food sensitivities are emerging in the literature and could be routinely incorporated into TK research platforms. The NIH’s 2020–2030 Strategic Plan for Nutrition Research [42] may involve TKs as de facto clinical research sites. Additionally, the recent White House Conference [18] includes recommendations for increased funding for research relating to nutrition and *Food Is Medicine* initiatives.

Regarding ***Food Is Medicine***, the aforementioned ***Food Is Medicine*** *Map* [35] goes beyond TKs and also includes Medically Tailored Meal Programs, Produce RX programs, Nutrition Incentive Programs and Fresh Food Farmacy Programs. Over time, these communities may collaborate on research to demonstrate how more coordinated access to healthy food options and educational programs could prevent disease, improve health outcomes and reduce health-care-related costs [43]. TKs can provide patient-centered care that goes beyond didactic education and offers the opportunity to address barriers to healthy eating for participants through clinic-to-community partnerships. TKs work adjunctively with Supplemental Nutrition Assistance Program (SNAP), SNAP Education (SNAP-ed), Women Infants and Children (WIC) and other federally funded food-related programs to improve nutrition security [44,45,46]. Additionally, TKs are meant to highlight patient-centered care to help guide participants through barriers while improving their food choices, as opposed to several of the other ***Food Is Medicine*** initiatives, which focus primarily on the provision of, or access to, health-enhancing foods.

### 2.8. Teaching Kitchens: Potential for Future Impact and Expansion

TKs have the potential to impact the education of health professionals [47], serve as translational research spaces and be evaluated in both medical and non-medical settings to reduce disease burden, health disparities and health care spending, while promoting a more environmentally sustainable food system through encouraging plant-forward diets which reduce greenhouse gases [6]. Importantly, TKs, when viewed as “shared community assets”, offer unique opportunities for the medical, biomedical, educational, public health, food, HR and business sectors to come together to enhance the health of people and the planet.

TKs are also being used in the setting of patients with physical and/or cognitive disabilities, as illustrated by the TK programs at the Harvard Spaulding Rehabilitation Center (https://spauldingrehab.org/education-training/cme/clinicians-chef-coaching, accessed on 10 June 2023) and the University of British Columbia’s Brain Health Center (https://teachingkitchens.org/member-listing/food-nutrition-health-program-faculty-of-land-and-food-systems/ [accessed on 10 June 2023]).

TKs to enhance the education and training of Health Professionals: As summarized in the recently passed House of Representatives Resolution [17], and as recapitulated in multiple publications [28,48,49,50,51], all licensed health professionals should be required to demonstrate minimal competencies involving nutrition recommendations in lay terms. Such recommendations should emphasize practical advice for patients with respect to (a) foods to consume more or less of and why—and how best to shop for these efficiently; (b) translation of nutrition science regarding the procurement and preparation of health-sustaining foods; and (c) knowledge on when and how to refer patients to a licensed nutrition professional. Consistent with the Academy of Nutrition and Dietetics’ Practice Guidelines, the more physicians understand about the critical role of nutrition in preventing and treating noncommunicable diseases, the more likely they are to collaborate with RDNs and refer patients for Medical Nutrition Therapy [52]. For more than 100 years, medical educators have required medical trainees to learn chemistry and anatomy, and recently, information technology in “laboratories” [53]. In this regard, TKs can be thought of as hands-on laboratories for this interprofessional nutrition education. This is not to replace the unique expertise of RDNs, but to equip other health professionals with a fundamental understanding of nutrition. Teaching health care professionals how to make healthier food choices in the absence of a TK is like trying to teach anyone about the benefits of swimming in the absence of a swimming pool.

TKs as Contributors to Future Research Networks and Sponsored Research Initiatives: Given the large number of TKs being built across a variety of sectors of society [5], research involving the impact of TK-related educational interventions may contribute to our understanding of innovative strategies whereby changes in diet and lifestyle-related behaviors can predictably lead to changes in biomarkers, clinical outcomes, behaviors and costs. This prospect aligns with the NIH’s 10-year Strategic Plan for Nutrition Research as it relates to Precision Nutrition and to the work of other NIH Institutes and Centers [42]; to the work of the CDC [54]; the actionable recommendations of the recently held White House Conference on Food, Nutrition and Health [18]; and the recent NIH Concept Clearance in support of Centers of Excellence on Food Is Medicine [55].

The Business Case for TKs as Shared Assets in An Era of Value-Based Care: Health care costs in the US were 17.8% of the gross domestic product (GDP) in 2021 and are expected to reach 20% of GDP by 2028 [56]. As the current fee-for-service reimbursement system is replaced by a value-based reimbursement approach [57], and as money is saved or made by optimizing health and well-being and keeping people out of the hospital, TKs may serve an important financial role in the context of future health care delivery systems—especially from the perspective of self-insured payers. Research aimed at testing the hypothesis that access to a TK program may alter behaviors, clinical outcomes, productivity and costs may gain support for future research in this area, supported by both the public and private sectors.

## 3. Limitations and Moving Forward

As noted above, there is still considerable variation across existing TKs in terms of their facilities, curricula and populations of interest. Additionally, research evaluating the impact of TKs on specific populations, including investigations aimed at addressing the needs of vulnerable populations, remains limited. However, in light of the recent article by Christopher Lynch, the Acting Director of the NIH Office Of Nutrition [58], describing the NIH’s approval in early 2023 of a concept clearance to establish Food Is Medicine Centers of Excellence, and the recently released NIH Request for Information (RFI) entitled “Food Is Medicine Research Opportunities” [59], it is likely that federally funded studies of *Food Is Medicine* and of TKs will soon increase in number, rigor and quality, with an increasing focus on vulnerable populations.

## 4. Conclusions

TKs serve as educational venues where individuals, families and communities can learn how to “eat, cook, move and think more healthfully”; are being built with increasing frequency across a range of settings as in-person or virtual classrooms and research laboratories; have the potential to improve self-care behaviors, biomarkers, clinical outcomes and costs; and may someday become valued shared assets of universities, health professional schools, hospitals, corporate worksites and communities. Well-conducted scientific investigations with large sample sizes are needed to estimate robust associations between participation in TKs and a range of behavioral, biological and economic outcomes. Efforts to assess their relevance to medical education, training, practice and cost containment deserve the collective attention of the interdisciplinary health professional community.

## Data Availability

Not applicable.
